# Poorly differentiated laryngeal neuroendocrine neoplasm with high serum calcitonin level, a case report, with literature review

**DOI:** 10.1002/ccr3.6751

**Published:** 2022-12-22

**Authors:** Maziar Motiee‐Langroudi, Athena Farahzadi, Reza Ansari, Hana Saffar, Rezvan Darabi, Mehrshad Abbasi

**Affiliations:** ^1^ Imam Khomeini Complex Hospital, Cancer Institute Tehran University of Medical Sciences Tehran Iran; ^2^ Division of Surgical Oncology, Cancer Institute Tehran University of Medical Sciences Tehran Iran; ^3^ Otorhinolaryngology Research Center Tehran University of Medical Sciences Tehran Iran; ^4^ Anatomical and clinical pathology, Cancer Institute, IKHC Tehran University of Medical Sciences Tehran Iran; ^5^ Resident of Internal Medicine, IKHC Tehran University of Medical Sciences Tehran Iran; ^6^ Department of Nuclear Medicine, Valiasr Hospital Tehran University of Medical Sciences Tehran Iran

**Keywords:** calcitonin, larynx, neuroendocrine tumor, primary.

## Abstract

Neuroendocrine neoplasm (NEN) of the larynx consists of 0.6% of laryngeal cancer and is the second most common type after squamous cell carcinoma (SCC). Laryngeal NEN rarely secret calcitonin and should be differentiated from medullary thyroid carcinoma. It makes a diagnostic and therapeutic challenge. We describe a case of a laryngeal NEN with calcitonin hypersecretion. A 59‐year‐old man presented to our clinic with recurrent cough, dysphonia, hoarseness, cervical mass, and significant weight loss. Diagnostic workup showed a supraglottic mass. Biopsy of the lesion revealed large‐cell neuroendocrine neoplasm. Further diagnostic workup showed elevated serum calcitonin level. The patient underwent total laryngectomy, thyroidectomy, and modified radical neck dissection. During his follow‐up, new subcutaneous nodules appeared that were biopsy‐proven metastases. Then adjuvant chemoradiotherapy was performed. Laryngeal NEN with hypersecretion of calcitonin is a rare entity. In patients with elevated serum calcitonin levels and head and neck tumors, it should be considered a differential diagnosis of medullary thyroid carcinoma. As the management and prognosis of these two neoplasms are entirely different.

## Abstract 2

Laryngeal NEN which consists of 0.6% of laryngeal cancer rarely secretes calcitonin and should be distinguished from medullary thyroid carcinoma. This poses diagnostic and therapeutic challenges as management and prognosis vary widely.

## 1 INTRODUCTION

Laryngeal neuroendocrine neoplasms (NEN) are a rare group of cervical NENs. Only about 500 cases were reported in the literature.^1^ They are the most common non‐squamous tumors of this organ and account for 0.6% of laryngeal neoplasm.^2^, ^3^ Laryngeal NENs are divided into epithelial and neural type tumors, the latter consisting of paraganglioma. Primary epithelial‐derived NEN is classified into three categories according to WHO blue book 2017: well‐differentiated carcinoma G1, moderately differentiated carcinoma G2, and poorly differentiated carcinoma G3.^1^ The last is divided additionally into two subtypes: small‐cell NEC (SmCNEC) and large‐cell NEC (LCNEC).^4^ Neuroendocrine carcinomas (NECs) in the larynx are usually positive for some cytokeratins and react to at least one neuroendocrine marker. They are characterized by neurosecretory granules and marker hormones, most commonly calcitonin.^5^

Laryngeal NENs rarely secret calcitonin. Eight cases of laryngeal NEN with calcitonin secretion are described in the literature.^1^ It has been theorized that calcitonin‐secreting NEN of the larynx represents ectopic thyroid medullary carcinoma due to laryngotracheal remnants of thyroid tissue.^6^

Laryngeal NENs are more common in men and have a strong association with heavy tobacco use. They mainly affect patients in the 5th to 7th decade of life. Supraglottis is the most common location. They can manifest as a polypoid or submucosal mass. They may ulcerate in higher‐grade tumors.^5^ Patients manifest with nonspecific symptoms primarily like hoarseness, sore throat, dysphagia, and rarely paraneoplastic syndromes due to hormone secretion.

Precise diagnosis of laryngeal NEN categories is essential because the natural history, treatment, and prognosis vary widely for each neoplastic one. Well‐differentiated carcinoma is uncommon and is treated with wide local excision, usually partial laryngectomy. Moderately differentiated tumors are more prevalent and more aggressive. Partial or total laryngectomy with elective or therapeutic neck dissection is the treatment of choice. Adjuvant chemo/radiotherapy may be beneficial in some cases. Both types of poorly differentiated neuroendocrine carcinomas are aggressive and should presume as metastatic. Irradiation and chemotherapy are used as treatments because surgery is ineffective. Local excision or partial laryngectomy is used to treat paragangliomas.^7^, ^8^

Calcitonin‐secreting neuroendocrine carcinomas of the supraglottic larynx are uncommon tumors. They should consider in patients with hypercalcitoninemia and head and neck tumors. The rarity of this entity makes it challenging to agree on treatment plans.^9^

We reviewed all the English published articles on laryngeal NEN with calcitonin secretion using databases such as Medline, PubMed, EMBASE, and the Cochrane Library until May 2022, and other related articles retrieved from those referenced in these papers. We found eight cases of laryngeal NEN with hypercalcitoninemia.

We report a rare case of NEN of the larynx with hypersecretion of calcitonin and lymph node involvement, which represents a diagnostic and therapeutic challenge.

### 1.1 Case presentation

A 59‐year‐old man presented to our clinic with recurrent cough for 1 year in addition to dysphonia and hoarseness for 6 months. He had a 4‐year history of enlarging right lateral cervical mass accompanied by 15 kg weight loss. He was a former opium addict. He had a history of hypothyroidism, COPD, and cardiac problems that had undergone CABG 2 years ago. His family history of neoplasm and endocrine disease were negative. On clinical examination, he was cachectic. There was a firm, fixed, non‐tender level II right cervical lymph node. The rest of the physical examination was normal.

**FIGURE 1:**
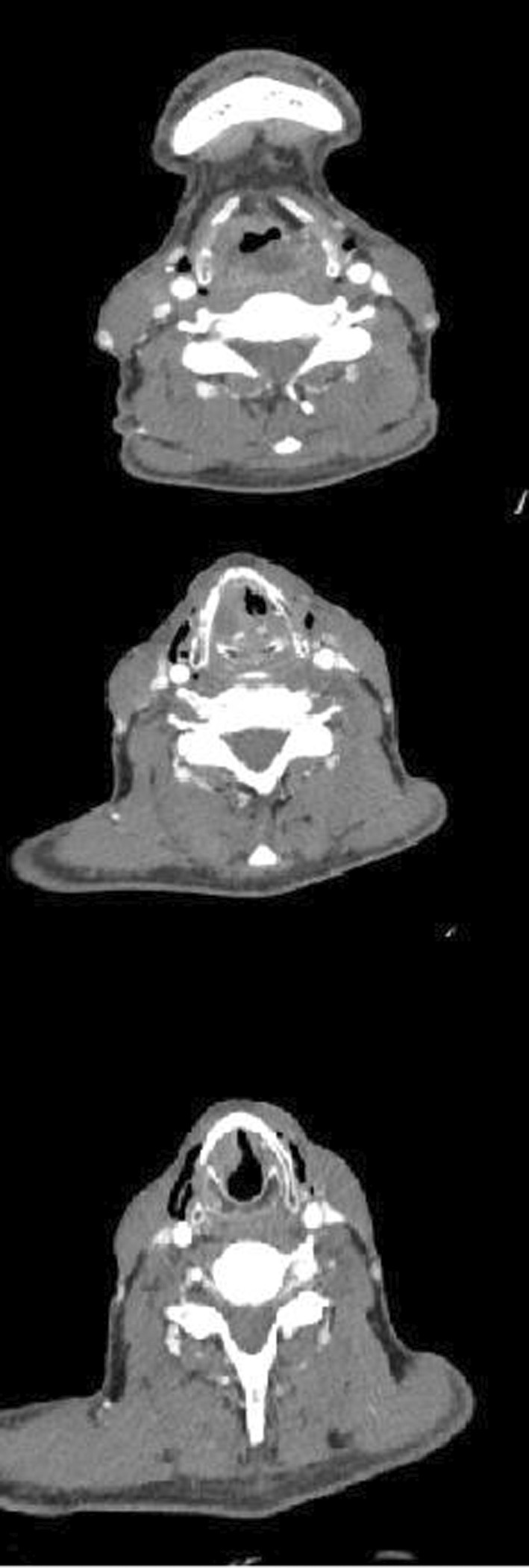
Spiral neck CT scan showed a heterogeneous supraglottic mass at midline with invasion to the left aryepiglottic fold, and inferior extension to glottis on the right side.

In additional diagnostic workup, a neck computerizing tomography (CT) scan with intravenous contrast was performed (Figure 1). It showed a heterogeneous supraglottic mass at midline with invasion to the left aryepiglottic fold, inferior extension to glottis on the right side, and right internal jugular vein chain lymphadenopathy.

**FIGURE 2:**
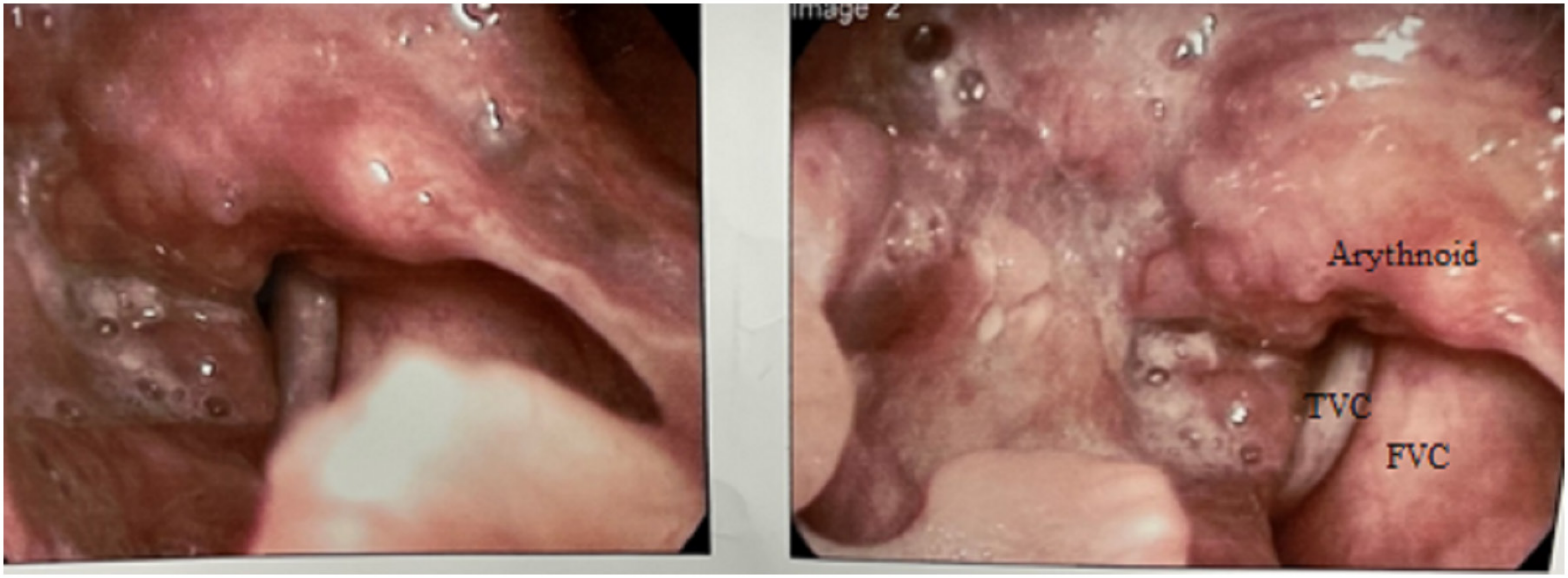
DLB showed a supraglottic tumor with involvement of right Hemi‐glottis and right piriformis sinus. Posterior cricoid was suspected.

**FIGURE 3:**
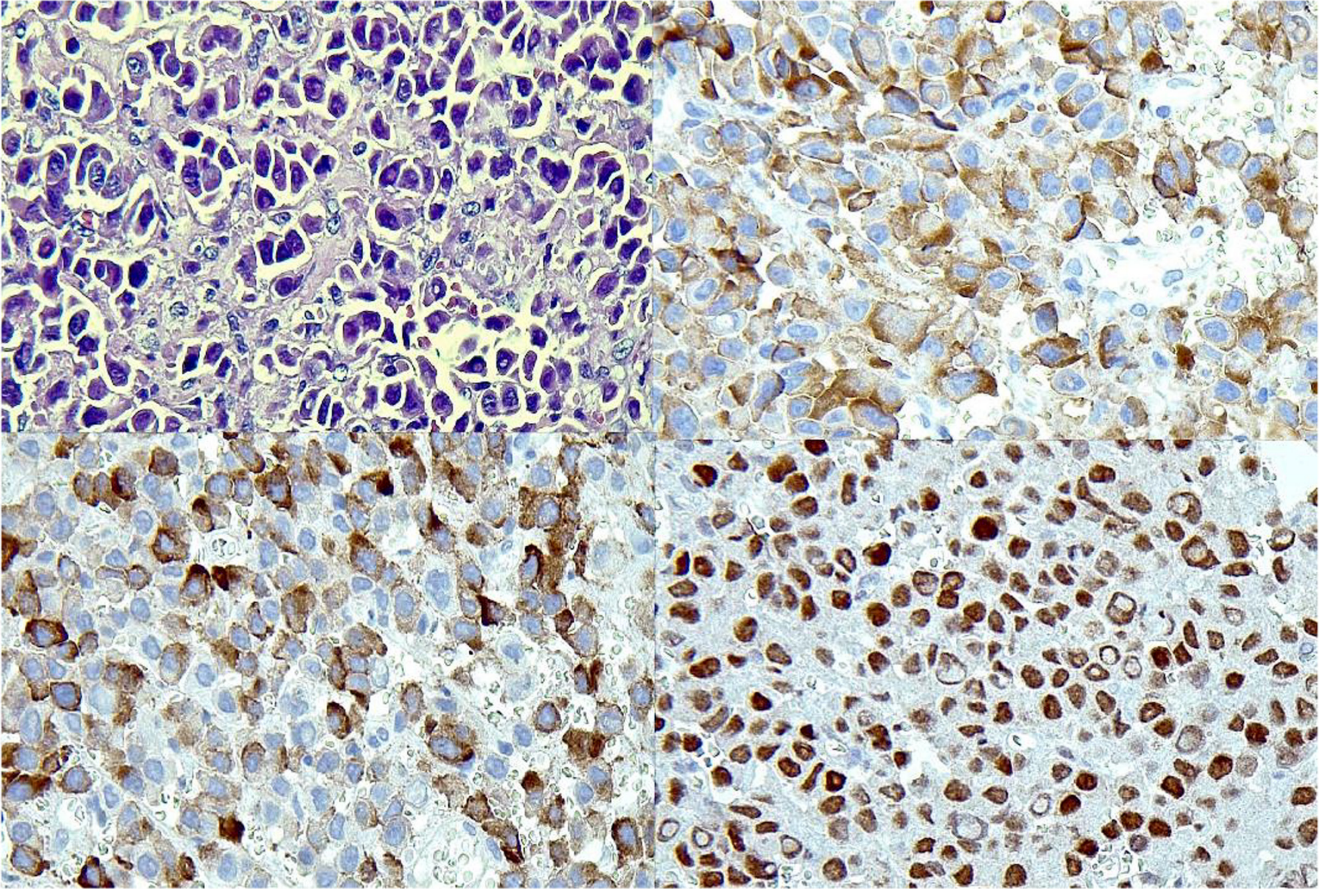
H&E stain, ×400. Synaptophysin IHC, ×400. Calcitonin IHC stain, ×400. TTF1 IHC stain, ×400

He underwent direct laryngoscopy and biopsy (DLB) and emergent tracheostomy due to his respiratory distress. DLB showed a supraglottic tumor with involvement of right Hemi‐glottis and right piriformis sinus. Posterior cricoid infiltration was suspected (Figure 2). A biopsy from the supraglottic lesion was performed. A microscopic examination from respiratory mucosa revealed infiltration of medium‐sized to large neoplastic cells with acidophilic cytoplasm, coarse chromatin pattern, and occasional prominent nuclei. The tumor exhibits areas of necrosis and a high mitotic rate (more than 10/mm^2^). Immunohistochemistry was positive for CKAE1/AE3, synaptophysin, CK7, TTF1, and Calcitonin (Figure 3). High‐grade neuroendocrine carcinomas of the larynx versus medullary thyroid carcinoma diagnosed. Serum calcitonin measurement was high (77 pg/ml), and serum CEA level was normal.

**FIGURE 4:**
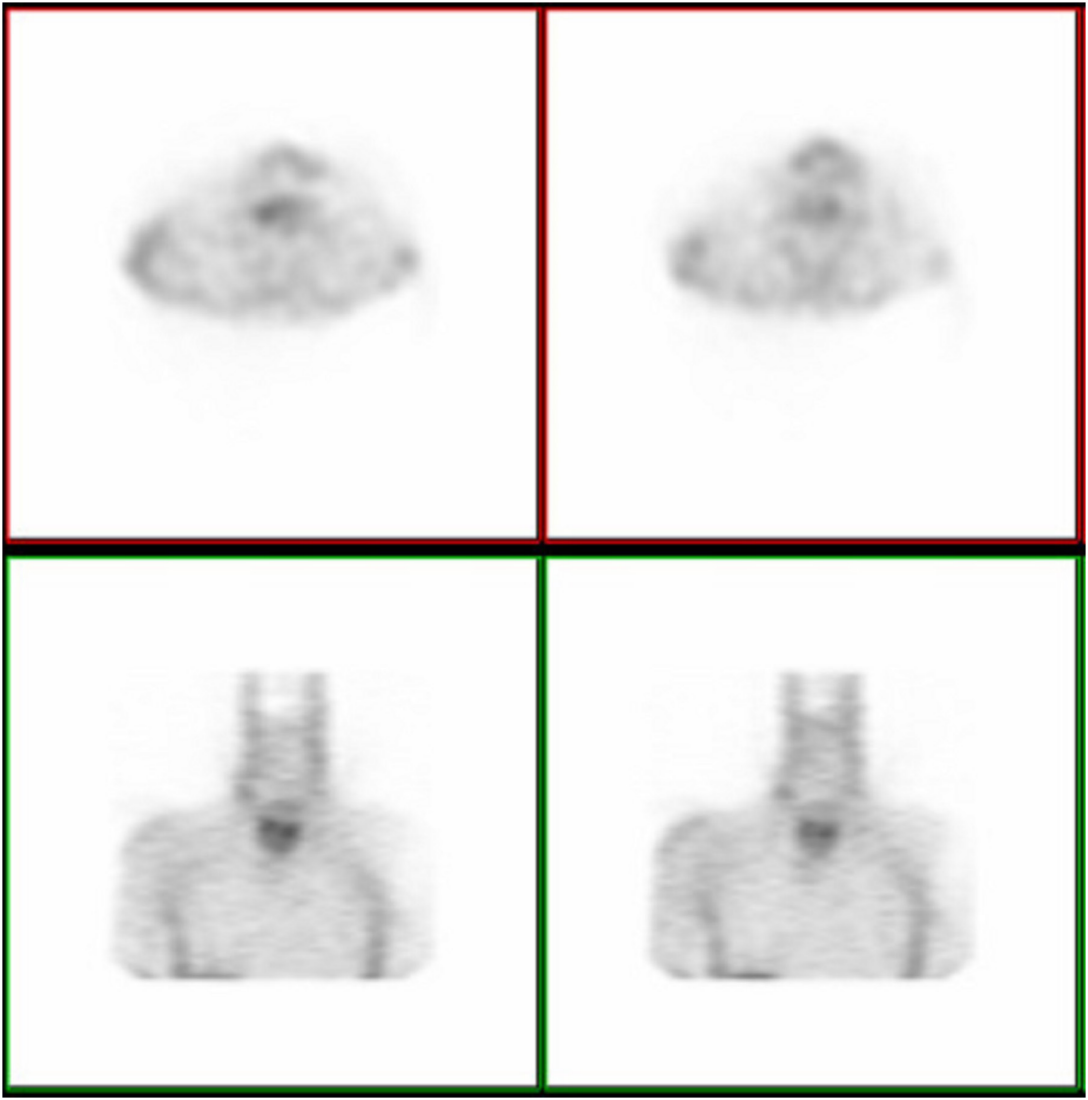
Octreotide Scintigraphy scan revealed an avid laryngeal tumor without evidence of regional or distant metastasis

**FIGURE 5:**
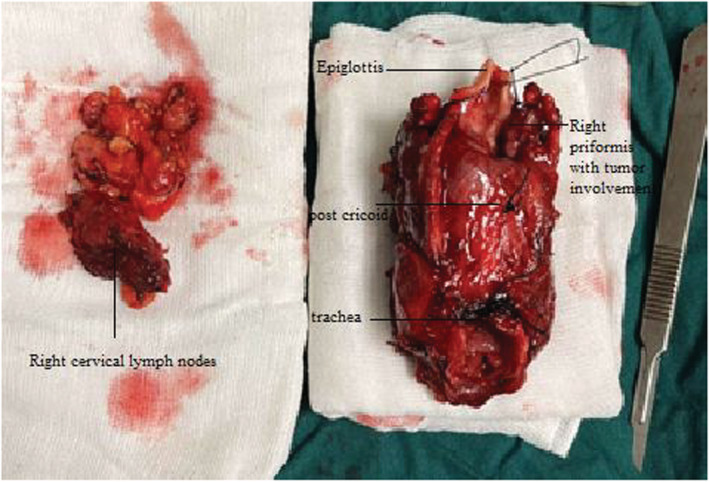
Intraoperative specimen of laryngectomy, TT, neck dissection

On further evaluation, an octreotide scintigraphy scan with 25 mCi ^99m^Tc pentetreotide was performed (Figure 4). It revealed an avid laryngeal tumor without evidence of regional or distant metastasis. The patient was introduced to the Multidisciplinary Tumor Board. Finally, we decided to perform a total laryngectomy, thyroidectomy, and right modified radical neck dissection. The patient then had surgery (Figure 5). The histological examination revealed a 3 cm multifocal high‐grade calcitonin‐positive neuroendocrine carcinoma of the larynx (large‐cell neuroendocrine neoplasm: LCNEC). The main bulk of the tumor was located in the medial aspect of the right pyriformis sinus, right TVC, and FVC. Also, there was a separate mass in the left pyriform sinus. All margins were free from tumor. The thyroid gland was submitted in total to pathology. Neither tumoral tissue nor C. cell hyperplasia was detected despite multiple recut sections. Five out of fifteen dissected lymph nodes were involved by the tumor. (mT3N2a).

The postoperative course was uneventful except for transient hypocalcemia that managed with oral calcium. The patient subsequently underwent adjuvant external beam radiotherapy to the larynx and the neck. His calcitonin level dropped to 17 pg/ml after 1 month of surgery. During 3 months of follow‐up, two subcutaneous nodules appeared in the scalp and back that were metastatic. Three suspected lesions in the bone scan were identified. Serum calcitonin level was 237. He has been undergoing chemotherapy.

## 2 DISCUSSION

We describe a rare case of laryngeal NEN with elevated serum calcitonin levels. While many neuroendocrine tumors secrete calcitonin, only seven cases of hypercalcitoninemia in NEN of the larynx have been reported.^1^, ^10^

Neuroendocrine tumors of the larynx are uncommon. Five hundred cases of neuroendocrine tumors of the larynx have been depicted in the literature since Goldman et al. first described them in 1969.^3^ Nevertheless, the larynx is the most prevalent site for head and neck neuroendocrine tumors.

They are found in the supraglottic, near the aryepiglottic fold, arytenoid, or false vocal cords in about 60 to 96 percent of cases.^4^, ^5^ Thyroid carcinomas, on the contrary, most commonly infiltrate the subglottic or trachea, leaving the supraglottic unaffected.^3^ Uncommon cases of medullary thyroid carcinoma (MTC) can invade the larynx and must distinguish from primary laryngeal NEN.^1^

Hoarseness, dysphagia, and sore throat are common nonspecific clinical symptoms associated with an obstructive mass lesion.^1^A paraneoplastic syndrome happens in rare cases when tumor cells produce abnormal hormones.^5^ Patients typically present with lymph node metastases in the neck and painful cutaneous metastases.^1^ In laryngeal neuroendocrine carcinomas, skin metastases are more common than in medullary thyroid carcinomas.^2^

In a case series of 20 laryngectomy specimens, non‐neoplastic neuroendocrine cells accounted for 5% of epithelial cells. They are located predominantly in the middle layer of the respiratory epithelium of the ventricle and subglottic area. NENs of the larynx are uncommon and account for 59 percent of non‐squamous carcinomas. Laryngeal NENs commonly have neuroendocrine histological and immunohistochemical traits. They may express chromogranin A, synaptophysin, and cytokeratins.^5^ Laryngeal NENs are frequently big cell carcinomas with stromal amyloid and TTF1 positivity and calcitonin staining, similar to MTC.^2^ Calcitonin immunostaining is usual in laryngeal NENs. The coexistence of the laryngeal NEN and elevated serum calcitonin levels are scarce.^1^

Medullary thyroid carcinoma (MTC) is a rare tumor of neuroendocrine origin that accounts for ∼3%–5% of all thyroid gland cancers. MTC originates from the C‐cells of the thyroid gland that secrete calcitonin. The epiglottis, laryngeal body, and superior parathyroid are originating from the mesenchyme of the fourth and sixth pairs of pharyngeal arches, where neural crest cells move before exhibiting C cell features. As a result, thyroid C cells and laryngeal neuroendocrine cells, which are parts of the neuroendocrine system, both express calcitonin and CEA.^1^

Both cancers stain strongly for synaptophysin, calcitonin, and CEA. It makes distinguishing NEN from MTC challenging. TTF‐1 has proven advantageous because it is significantly and diffusely positive in MTC but commonly negative or modestly positive in NEN.^11^, ^12^ It can express in variable ways and can be strongly positive in some cases like ours.^13^ In MTC, serum CEA and serum calcitonin are almost always high, unlike NEN.^3^ In our case, TTF1 was strongly positive, serum calcitonin level was high, and serum CEA was normal. Previous cases of hypercalcitoninemia in laryngeal NEN are summarized in Table 1.

**TABLE 1:** Summarize cases with laryngeal NENs with calcitonin secretion

References	Organ‐histology	Age	Sex	Symptoms	Value (peak after Stimulation Test)	Normal range	Treatment	Post‐treatment CT	Outcome	Immunostaining
Sweeney et al. (1981)	Larynx—poorly differentiated NEN (Left arytenoid, three cervical lymph nodes)	54	M	Hoarseness	1200 pg/ml (1500)	0–200	Laryngothyroidectomy	NA	NA	Calcitonin + CEA + TTF − 1—no report
Smets et al. (1990)	Larynx—atypical carcinoid (Epiglottis, three submandibular lymph nodes, skin, brain)	55	M	Hoarseness and Dysphagia	3790 pg/ml	0–100	RT + CHT + left neck dissection; total laryngectomy for recurrence; CHT for metastasis	NA	After 3 years DOD	Calcitonin + CEA + TTF − 1—no report Cytokeratin + Chromogranin A + NSE+.
Insabato et al. (1993)	Larynx—moderately differentiated NEN (Right arytenoid.)	69	M	Hoarseness	970 pg/ml	0–300	Partial laryngectomy + RT; surgery for subsequent local recurrence	45 pg/ml (rising to 1210 pg/ml after recurrence.	Free of disease	Calcitonin + CEA—no report TTF − 1—no report Cytokeratin + Chromogranin A + NSE+
Machens A et al. (2000)	Larynx—moderately differentiated large‐cell NEN (Right arytenoid)	61	M	NA	16.3 mg/ml (29.2)	1–10	Partial laryngectomy, RT, subtotal Thyroidectomy	NA	After 32 months DOD	NA
Chung JH et al. (2004)	Larynx–moderately differentiated NEN (Epiglottis, cervical lymph nodes, skin nodules in the right arm, bones)	57	M	Hoarseness, dysphagia, voice change, foreign‐body sensation in the throat.	Elevated (after metastasis: 599 pg/ml)	NA	Supraglottic laryngectomy, neck dissection, CHT	NA	After 27 months DOD	NA
LaBryer et al. (2015)	Larynx—poorly differentiated adenocarcinoma t with neuroendocrine features (Right arytenoid, 7 cervical lymph nodes, and thyroid)	57	M	Painful neck mass, otalgia, odynophagia, and hoarseness	157 pg/ml	0–8	Total laryngectomy, bilateral neck dissection + RT + Total thyroidectomy	35 pg/ml, Metastasis appearance after 6 months (calcitonin increased to 320 pg/ml	NA	Calcitonin + CEA + TTF – 1 + (focally) Cytokeratin + Chromogranin A + NSE—not done
Hanadi Fatani et al. (2016)	moderately differentiated NET of Larynx (right epiglottis, right level II cervical lymph nodes)	51	F	Sore throat, change in voice, mild odynophagia, left ear pain, dysphagia	N/A	N/A	Laser resection of epiglottis, total thyroidectomy with bilateral modified radical neck dissection, Adjuvant external beam radiotherapy	12 (<8)	Disease free after 2 years	CKpan+, Synaptophysin+,Chromogranin A+, CEA+,calcitonin+,TTF‐1‐
Feola T et al. (2020)	Larynx—moderately differentiated NEN (Epiglottis, 2 cervical lymph nodes, skin)	59	M	Neck mass, painful subcutaneous nodules	50 pg/ml (56.6)	0–10	Total thyroidectomy, lymphnode dissection, skin metastases excision, OCT 30 mg/28 days and Everolimus 10 mg/day	NA	Partial response after 15 months follow‐up	NA
Present study	Large‐cell neuroendocrine carcinoma of the larynx (right pyriformis sinus, right TVC and FVC, left pyriform sinus, cervical lymph nodes)	58	M	Recurrent cough, dysphonia, hoarseness, right lateral cervical mass	77 pg/ml	0.2–27	Total laryngectomy, Total thyroidectomy, selective neck dissection	17	New subcutaneous Nodules after 3 months	CKAE1/AE + CK7 + TTF + synaptophysin+

Abbreviations: CEA, carcinoembryonic antigen; CHT, chemotherapy; DOD, died of disease; M, male; NA, not evaluated; NEN, neuroendocrine neoplasm; NSE, neuron‐specific enolase; RT, radiotherapy; TTF1, thyroid transcription factor1.

Sweeney et al.^14^ reported the first case of a laryngeal NEN with hypercalcitoninemia in the absence of a primary thyroid tumor in 1981.

The categorization of laryngeal neuroendocrine carcinomas has been adjusted and corrected to recognize specific entities' diverse biological behavior and histological properties. The main controversy is large‐cell neuroendocrine carcinoma. It initially was classified as atypical carcinoid/moderately differentiated NEC, grade II. It is now defined as a poorly differentiated NEC, grade III. The WHO Blue Book 2017 classifies neuroendocrine tumors into three types: well‐differentiated, moderately differentiated, and poorly differentiated.^4^, ^6^ Our patient was in the poorly differentiated group.

The most recent meta‐analysis included 436 cases of laryngeal NECs, consisting of 23 well‐differentiated tumors, 163 moderately differentiated tumors, 183 SmNECs, 29 LCNECs, and 38 unidentified carcinoid tumors. Except for well‐differentiated NEC, which showed no gender preference, males were more frequently affected than females (3:1), mostly in their fifth to seventh decades of life. Laryngeal NEN has a significant link to heavy cigarette smoking. Recent research has found that poorly differentiated laryngeal NECs are not related to high‐risk HPV infections.^3^, ^4^, ^6^

The integration of clinical, biochemical, and radiological data is used to differentiate between MTC and laryngeal NEN.

Increased serum calcitonin level predicts medullary thyroid carcinoma (MTC) while not a pathognomonic indicator. Renal insufficiency, hyperparathyroidism, neuroendocrine neoplasms (NENs), and non‐neuroendocrine carcinomas (lung, colon, breast, and prostate carcinomas) can all raise serum calcitonin levels, as can some medicines.^2^

Calcitonin‐secreting NENs can be differentiated from C‐cell diseases by their lack of response to the stimulation test. Specific cutoffs for stimulated calcitonin have not been proposed in the literature. Calcitonin values in calcitonin‐secreting NENs after Pentagastrin stimulation have only been recorded in a few case reports and small case series. The measurement of basal and stimulated calcitonin should be included in the differential diagnosis of thyroid and extra‐thyroid NENs, to avoid unnecessary thyroidectomies. More research is needed to determine particular cutoffs.^1^, ^2^, ^9^

Because the natural history, therapy, and prognosis of the various neoplastic groups differ widely, precise identification of tumor type is critical. Each tumor has a different outcome and management. Surgery is usually utilized for all tumor types, with chemotherapy added for moderately differentiated and small or large‐cell neuroendocrine carcinomas. Recurrences are common in patients who have an advanced illness.^5^

Well‐differentiated NEN is uncommon. It is treated with a large local excision, usually a partial laryngectomy, and no neck dissection. Partial or total laryngectomy with elective or therapeutic neck dissection is used to manage moderately differentiated NEN tumors. In some circumstances, adjuvant chemo/radiotherapy may be beneficial. Patients with small‐cell or large‐cell neuroendocrine carcinoma advantage more from chemoradiotherapy.^7^ Irradiation and chemotherapy are used as treatments for poorly differentiated ones because surgery is ineffective.^8^

Well‐differentiated NEC (carcinoid) had a 5‐year disease‐specific survival rate of 100%. However, according to some studies due to their rarity and misconception in the literature about their atypical counterparts, determining meaningful survival statistics for typical carcinoids is challenging. They have a higher tendency for metastases and thus a worse prognosis compared to older studies.^8^ Moderately differentiated NEC (atypical carcinoid), SmCNEC, and LCNEC had a 5‐year disease‐specific survival rate of 53%, 19%, and 15%, respectively.^4^, ^7^ Most LCNEC patients develop distant metastases and die within 2 years.^15^ Laryngeal paraganglioma has a generally benign biological behavior and a good prognosis.^8^

In our patient, total laryngectomy, modified radical neck dissection, and total thyroidectomy (to pathologically exclude medullary thyroid carcinoma) were performed after consulting with a multidisciplinary tumor board. He received adjuvant chemoradiotherapy for his new onset subcutaneous metastatic nodules.

In conclusion, the differential diagnosis in a patient with head/neck cancer and hypercalcitoninemia must consist of MTC and neuroendocrine tumors. Due to significant overlap in features, even pathological diagnosis may be challenging. Serum CEA levels and staining patterns for TTF‐1 may help differentiate these two tumor types. This case report and the corresponding literature review provide useful insight for clinicians to improve their knowledge in the diagnosis and management of this rare and aggressive neoplasm.

## AUTHOR CONTRIBUTIONS

**Maziar Motiee‐Langroudi:** Conceptualization; investigation; methodology; project administration; supervision; writing – review and editing. **Athena Farahzadi:** Conceptualization; data curation; formal analysis; investigation; methodology; project administration; resources; software; validation; visualization; writing – original draft; writing – review and editing. **Reza Ansari:** Data curation; investigation; writing – review and editing. **Hana Saffar:** Writing – original draft; writing – review and editing. **Rezvan Darabi:** Writing – review and editing. **Mehrshad Abbasi:** Writing – review and editing.

## FUNDING INFORMATION

None.

## CONFLICT OF INTEREST

None.

## ETHICAL APPROVAL

The patient/participant provided their written informed consent to participate in this study.

## CONSENT

Written informed consent was obtained from the patient to publish this report in accordance with the journal's patient consent policy.

## Supporting information


Appendix S1

## Data Availability

The data that support the findings of this study are available on request from the corresponding author. The data are not publicly available due to privacy or ethical restrictions.
