# Supplementary material for: Poorly differentiated laryngeal neuroendocrine neoplasm with high serum calcitonin level, a case report, with literature review
Source: Clin Case Rep. 2022 Dec 22;10(12):e6751. doi: 10.1002/ccr3.6751 (PMC9780421; doi:10.1002/ccr3.6751)
Supplement: Supplementary file 1 — Appendix S1 [file CCR3-10-e6751-s001.docx]

**Timeline**

Recurrent cough since last year

Dysphonia, hoarseness since 6 months ago

DLB and emergent tracheostomy 2 months ago; biopsy neuroendocrine carcinoma

adjuvant chemotherapy

Adjuvant radiotherapy

Total laryngectomy, total thyroidectomy, selective neck dissection

15 kg weight loss during one year

Symptom-free for about 3 months when two subcutaneous nodules appeared. Calcitonin level elevated. Three suspected bone lesions were found.

Histology examination: calcitonin positive large cell neuroendocrine carcinoma of larynx (T3N2a)

Cervical CT scan: supraglottic mass

Octreotide scintigraphy scan: laryngeal avid mass
